# Cell models of arrhythmogenic cardiomyopathy: advances and opportunities

**DOI:** 10.1242/dmm.029363

**Published:** 2017-07-01

**Authors:** Elena Sommariva, Ilaria Stadiotti, Gianluca L. Perrucci, Claudio Tondo, Giulio Pompilio

**Affiliations:** 1 Vascular Biology and Regenerative Medicine Unit, Centro Cardiologico Monzino-IRCCS, via Parea 4, Milan 20138, Italy; 2 Department of Clinical Sciences and Community Health, Università degli Studi di Milano, Via Festa del Perdono 7, Milan 20122, Italy; 3 Cardiac Arrhythmia Research Center, Centro Cardiologico Monzino-IRCCS, via Parea 4, Milan 20138, Italy

**Keywords:** Arrhythmogenic cardiomyopathy, ACM, ARVC, Cell models, *In vitro*, Molecular mechanisms

## Abstract

Arrhythmogenic cardiomyopathy is a rare genetic disease that is mostly inherited as an autosomal dominant trait. It is associated predominantly with mutations in desmosomal genes and is characterized by the replacement of the ventricular myocardium with fibrous fatty deposits, arrhythmias and a high risk of sudden death. *In vitro* studies have contributed to our understanding of the pathogenic mechanisms underlying this disease, including its genetic determinants, as well as its cellular, signaling and molecular defects. Here, we review what is currently known about the pathogenesis of arrhythmogenic cardiomyopathy and focus on the *in vitro* models that have advanced our understanding of the disease. Finally, we assess the potential of established and innovative cell platforms for elucidating unknown aspects of this disease, and for screening new potential therapeutic agents. This appraisal of *in vitro* models of arrhythmogenic cardiomyopathy highlights the discoveries made about this disease and the uses of these models for future basic and therapeutic research.

## Abstract (precis)

**Summary:** *In vitro* models of ACM provide insights into the molecular mechanisms of this disease. This reappraisal offers a comprehensive vision of past discoveries and constitutes a tool for future research.

## Introduction

**Fig. 1.**
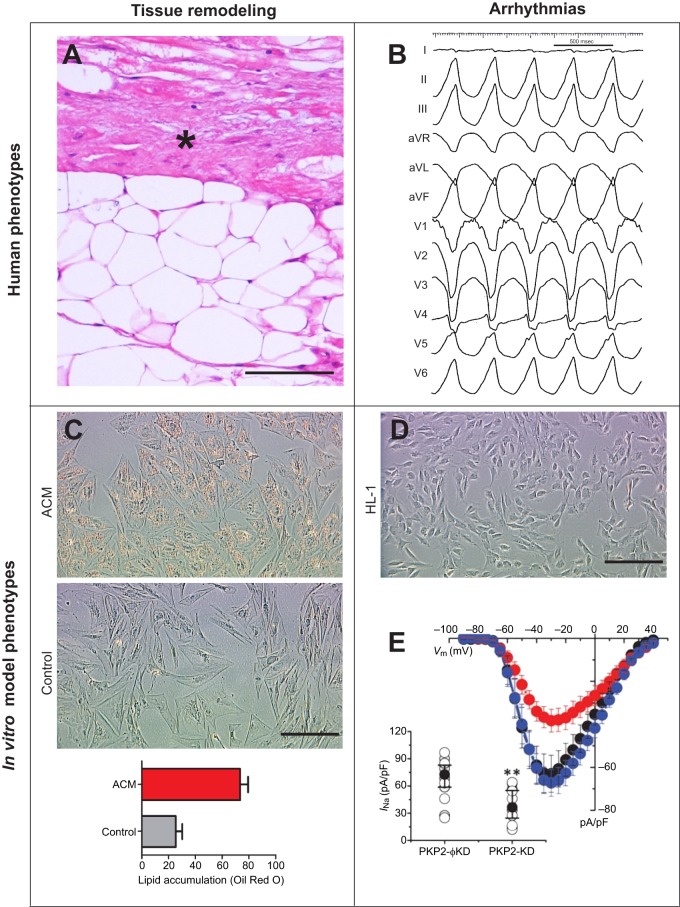
**Pathogenic cardiac changes in ACM.** (A,B) Representative images of key phenotypic features in human ACM. (A) Hematoxylin–eosin staining of the right ventricle (RV) in an ACM patient's heart shows fat deposits (white areas) and disarrayed cardiomyocyte architecture with fibro-fatty infiltrations (asterisk). (B) An electrocardiogram trace (12 leads, listed on the left-hand side, representing the electrical activity from electrodes on the body surface) of a typical RV arrhythmia, which commonly occurs in ACM patients. (C-E) Representative light microscopy images of ACM *in vitro* models and studies of fat accumulation and arrhythmias. (C) Oil Red O staining of isolated cardiac mesenchymal stromal cells (C-MSCs) from an ACM patient and control highlights the typical lipid accumulation seen in ACM. Lipid accumulation is measured by evaluation of red areas into the cell lipid droplets. (D) Depiction of murine HL-1 cells, which can be used to generate *in vitro* models for electrophysiological studies of the sodium channel Nav1.5 in ACM. (E) The main graph depicts, on the *y*-axis, the average peak of sodium current (*I*_Na_) density [measured in picoamperes/picofarad (pA/pF)] and, on the *x*-axis, voltage max [*V*_m_; measured in millivolt (mV)] in wild-type (WT) HL-1 cells (black trace), HL1 cells treated with *PKP2*-silencing construct (PKP2-KD; red trace) and HL-1 cells treated with a non-silencing construct (PKP2-φKD; blue trace). The corresponding dot plot, in the inset, shows that silencing *PKP2* in HL-1 cells (PKP2-KD) leads to a statistically significant (**P<0.005) decrease in sodium current density (*I*_Na_) (see main text for a discussion of the effect of PKP2 loss on sodium current). Adapted with permission from Cerrone et al., 2014. This image (E) is not published under the terms of the CC-BY licence of this article. Promotional and commercial use of the material in print, digital or mobile device format is prohibited without permission from the publisher Wolters Kluwer. Please contact healthpermissions@wolterskluwer.com for further information. Scale bars: 100 μm.

Arrhythmogenic cardiomyopathy (ACM) is a genetic disease associated with ventricular arrhythmias and a high risk of sudden cardiac death (see Box 1 for a glossary of terms). ACM affects mainly young individuals and trained athletes, and has a worldwide prevalence ranging from 1:1000 to 1:5000 (Basso et al., 2009). ACM is characterized by the substitution of the myocardium, the heart muscle, with fibro-fatty deposits, particularly within the free wall of the right ventricle (RV) (Fig. 1). This process exacerbates electric instability and causes impaired ventricular mechanical function, leading to arrhythmias and progressive heart failure (Box 1) (Marcus et al., 1982).

**Box 1. Glossary**

**Aneurismal dilation:** distention of the ventricular wall, interfering with correct myocardium contractility.

***Area composita*:** cardiac mixed-type junction composed of both desmosomal and adherens junctional proteins.

**Auricle:** small conical pouch projecting from the upper anterior portion of each atrium.

**Brugada syndrome:** a genetic disease mainly caused by mutations affecting sodium channels that lead to a reduced flow of sodium ions (Na^+^) into cardiac cells, which alters beating of the heart. It is characterized by ECG alterations and an increased risk of sudden cardiac death.

**Cardiac excitation–contraction coupling:** sequence of reactions, starting from electrical impulse, which drives intracellular calcium release; calcium binds troponin and activates muscle contraction.

**Current density of channels:** the amount of current passing through a cross-sectional area of a conductor in a specific time window.

**Digenic/compound heterozygosis:** co-inheritance of disease alleles of different genes (digenic) or of the same gene (compound).

**ECG:** electrocardiogram; the graphic reproduction of electrical activity of the heart, recorded from the body surface. The ECG trace is composed of different traits, named waves:

• **P wave:** wave corresponding to the depolarization of the atria.

• **QRS complex:** the series of waves representing ventricular depolarization (**Q:** septum; **R:** left ventricle apex; **S:** basal and rear regions of left ventricle). The complex duration is between 60 and 90 ms.

• **ε wave:** a small positive deflection buried in the end of the QRS complex. It represents delayed activation of affected areas of the right ventricle.

• **T wave:** corresponds to the repolarization of the ventricles.

**Heart** **failure:** a broad spectrum of heart impairments, leading to less-efficient blood pumping.

**Hippo pathway:** signaling pathway involved in the regulation of cellular proliferation, apoptosis and self-renewal. The core Hippo pathway consists of a kinase cascade leading to the inhibition of nuclear translocation of two main transcriptional factors, YAP and TAZ. In ACM, Hippo kinases are activated, YAP is phosphorylated and its canonical transcriptional activity inhibited.

**Implantable cardiac defibrillator (ICD):** device to perform cardioversion/defibrillation in the case of ventricular tachycardia, which could otherwise possibly lead to ventricular fibrillation and sudden death.

**Intercalated disc:** specialized intercellular cardiomyocyte area providing structural and functional connection between adjacent cardiomyocytes. It includes gap junctions, fascia adherens, desmosomes, ion channels and mechanoreceptors.

**Left/right bundle branch block:** pathological condition in which a delay or obstruction occurs along the conduction paths of electrical impulses to the left or the right cardiac ventricle, respectively.

**Lipothymia:** fainting or a feeling of faintness.

**Long QT syndrome:** a rare congenital disorder of delayed repolarization of the heart (prolongation of the QT interval on ECG), leading to a higher risk of ventricular arrhythmias, ventricular fibrillation and sudden death.

**Myocardial remodeling:** alteration in the structure (dimensions, mass, shape) of the heart. Specifically, in ACM the main features are fibrous and fatty deposits in the ventricular myocardium, with thinning of the free walls and segmental or diffuse atrophy.

**Palpitation:** perception of rapid and/or irregular heartbeats.

**Second heart field:** area of multipotent progenitor cells contributing to myocardium formation during heart development.

**Sudden cardiac death:** unexpected natural death owing to cardiac arrest, heralded by abrupt loss of consciousness within 1 hour of the onset of acute symptoms.

**Syncope:** partial or complete loss of consciousness due to a temporary reduction in blood flow and shortage of oxygen to the brain.

**Ventricular arrhythmias:** abnormal rapid heart rhythms originating from the ventricles.

**Wall motion abnormalities:** abnormal motion of a region of the heart muscle, causing heart dysfunction. It can be reduced (hypokinesia), absent (akinesia) or increased (hyperkinesia).

**Wnt pathway:** signaling pathway involved in various cellular process, such as adhesion, self-renewal, differentiation, migration and proliferation. Wnt proteins are glycoproteins that act as ligands of Frizzled receptors. The active pathway is characterized by the inhibition of glycogen synthase kinase 3, which allows the translocation of cytosolic β-catenin into the nucleus and the subsequent transcription of specific genes. In ACM, plakoglobin is thought to compete with β-catenin, activating adipogenesis, fibrosis and apoptosis.

ACM is mostly inherited as an autosomal dominant trait and is characterized by incomplete penetrance and variable expressivity (Basso et al., 2009). Recessive forms and the contribution of digenic and compound heterozygosis (Box 1) have also been reported (McKoy et al., 2000; Norgett et al., 2000; Soveizi et al., 2017; Xu et al., 2010). Despite genetic heterogeneity, the majority of genotyped ACM patients harbor mutations in genes that encode desmosomal proteins (Box 2), including plakoglobin (*JUP*), desmoplakin (*DSP*), plakophilin-2 (*PKP2*), desmoglein-2 (*DSG2*) and desmocollin-2 (*DSC2*) (Lazzarini et al., 2015). Mutations in non-desmosomal genes, including transforming growth factor-β3 (*TGFB3*), ryanodine receptor 2 (*RYR2*), transmembrane protein 43 (*TMEM43*), lamin A/C (*LMNA*), desmin (*DES*), titin (*TTN*), phospholamban (*PLN*) and αT-catenin (*CTNNA3*), are also proposed to associate with ACM (Lazzarini et al., 2015), although phenotypical overlap with other cardiomyopathies cannot be excluded.

**Box 2. Desmosome**

Desmosomes (pictured below) are intercellular junctions that provide strong adhesion between cells. They contain three major components: desmoplakin, which binds cytoskeleton intermediate filaments, transmembrane proteins (desmocollin 2 and desmoglein 2), and armadillo proteins (plakoglobin and plakophilin 2), which mediate the interactions between transmembrane proteins and desmoplakin. In the heart, this protein network gives mechanical strength, providing stability and integrity to the cardiac structure, contributes to tissue morphogenesis and development, and it also plays an important role in regulating crucial aspects of cell behavior, such as cell proliferation and differentiation.

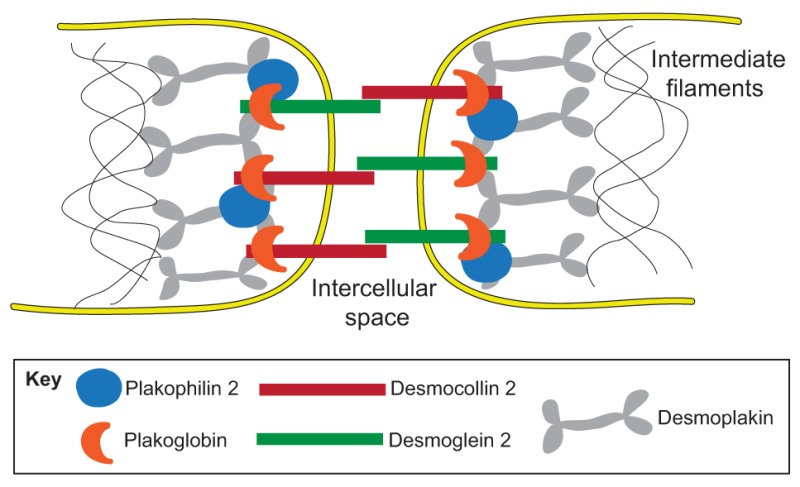

In the clinic, ACM presents a wide phenotypic spectrum (Box 3). Despite having genetic bases, ACM is not a congenital disease; clinical manifestations often develop between the second and fourth decade of life (Nava et al., 2000). Moreover, despite the autosomal inheritance, more males are clinically affected than are females (Corrado and Thiene, 2006; Bauce et al., 2008). Indeed, males show earlier arrhythmia manifestation (Bhonsale et al., 2013) and more severe disease expression (Bauce et al., 2008; Marcus et al., 2007, 1982; Blomstrom-Lundqvist et al., 1987; Merner et al., 2008). Moreover, frequent and competitive exercise increases the risk of malignant arrhythmias, heart failure and sudden death in ACM gene-mutation carriers (Corrado et al., 1990).

**Box 3. Clinical features of ACM**

ACM is broadly characterized by the presence of fibro-fatty deposits in the myocardium and by arrhythmia, although it is a phenotypically heterogeneous condition (Corrado et al., 1997). About 50% of patients show malignant ventricular arrhythmias at onset, with palpitations, lipothymias and syncopes. The frequency of ventricular arrhythmias commonly correlates with the severity of myocardial alterations (Hulot et al., 2004; Pinamonti et al., 2011). Notably, ventricular arrhythmias are often elicited by physical activity, during sympathetic nervous system activation (Wichter et al., 2000). On assessment by electrocardiogram (ECG), arrhythmias show a left bundle branch block with superior axis morphology, owing to their origin from the cardiac area called ‘ACM triangle’ (RV inflow tract, outflow tract, apex) (Marcus et al., 2010). In rare cases, mostly in the presence of mutations in the *DSP* and *DSC2* genes, a predominant left ventricular (LV) degeneration (Navarro-Manchon et al., 2011; Sen-Chowdhry et al., 2008; Bauce et al., 2010) is present, with right-bundle-branch-block-associated ventricular arrhythmias (Romero et al., 2013).

Commonly, sudden cardiac death occurs as first manifestation of the disease, even without overt cardiac structural abnormalities (Sen-Chowdhry et al., 2005). Conversely, in asymptomatic patients, ACM is suspected if particular ECG abnormalities are seen, such as T-wave inversion, QRS duration above 110 ms, prolonged rise of S wave and the presence of an ε wave (Iyer and Chin, 2013). To prevent sudden death due to ventricular arrhythmias, often an ICD is implanted.

As the disease worsens, cardiac structural alterations appear caused by a progressive replacement of the RV myocardium with fibro-adipose tissue, starting from the epicardium and extending transmurally to the endocardium and commonly diffusing into the LV. This myocardial atrophy causes aneurysmal dilation and wall motion abnormalities and leads, in advanced stages, to right- or bi-ventricular severe heart failure (Corrado et al., 1997; Romero et al., 2013) and eventually to heart transplant (Corrado et al., 2015). The fibro-adipose replacement creates areas of electrically inert tissue, which interferes with electrical impulse conduction and contributes to the typical ECG features and to the malignant arrhythmias of the disease. This fibro-adipose replacement of myocardial tissue is considered the hallmark of ACM when associated with myocyte degeneration and inflammation (Burke et al., 1998). See the Glossary (Box 1) for definitions of clinical terms used in this Box.

Lifestyle modifications, anti-arrhythmic drugs, implantable cardioverter defibrillator (ICD; Box 1) and eventually heart transplantation are the currently available therapeutic options for treating ACM. However, to date, a therapy that can tackle the cause of this disease is not available.

ACM is a relatively newly recognized disease, having only being described in 1977 as a distinct clinical entity (Fontaine et al., 1977). Since then, significant advances have been made in understanding its etiology and pathogenesis, and in diagnosing and managing the disease. Nevertheless, several biological and clinical features of ACM remain to be elucidated. Here, we review what is currently known about the etiology of ACM and its molecular mechanisms, focusing on *in vitro* models that are helping researchers unravel the pathology of this disease and to test hypotheses concerning its etiology and treatment. To date, a systematic review of ACM cell models, their strengths and limitations, and the insights into disease pathogenesis that they have provided, was lacking. For *in vivo* model reappraisal, we refer the reader to earlier reviews (McCauley and Wehrens, 2009; Lodder and Rizzo, 2012; Pilichou et al., 2011).

## ACM etiopathogenesis: the theories

The origin of ACM is still largely unknown, but different theories have been advanced to explain its etiology. In the disontogenic hypothesis, now abandoned, ACM was believed to be a congenital defect that arose from abnormal embryonic development of the RV. This explains why Fontaine and colleagues called the disease arrhythmogenic right ventricular dysplasia (ARVD) (Fontaine et al., 1977). Actually, the developmental problems are typical of Uhl's disease, a condition characterized by the complete absence of the parietal wall of the RV and which has, in the past, been confused with ACM (Uhl, 1952). Now the two diseases are recognized as different clinical conditions. Uhl's disease is usually diagnosed in neonatal or infant life, whereas ACM patients typically manifest symptoms from adolescence. Moreover, myocardial fibro-adipose replacement is not present in Uhl's anomaly (Pamuru et al., 2010). Therefore, the term ‘dysplasia’ was replaced with ‘cardiomyopathy’ (ARVC), which better describes the disease (Basso et al., 2010). Since the description of biventricular and left-dominant forms (Saguner et al., 2014), the name has been updated to arrhythmogenic cardiomyopathy (ACM).

The inflammatory theory was proposed to address the origin of the inflammatory cells found in the myocardium of ACM patients. It is not clear whether the inflammation occurs as a consequence of cardiomyocyte death or as a primary infective/immune mechanism. The presence of cardiotropic viruses (e.g. enterovirus and adenovirus) has indeed been reported in the ACM myocardium (Bowles et al., 2002). Further studies are needed to unravel the relevance of inflammation in this disease pathogenesis.

The cardiomyocyte transdifferentiation hypothesis was advanced to give an explanation of the phenomenon of myocardial substitution with fibro-fatty deposits (d'Amati et al., 2000). It is based on the supposition that the cardiomyocytes in ACM hearts could reprogram and differentiate into adipocytes as a consequence of the genetic defect. However, it is challenged today because of the limited evidence of the de-differentiation capabilities of adult cardiomyocytes.

The dystrophic theory of ACM origin currently prevails because of the significant similarities of ACM with skeletal muscular dystrophies (Basso et al., 1996). According to this hypothesis, the fibro-fatty deposits in ACM myocardium are considered to be scar tissue that replaces dead cardiomyocytes. In line with this, ACM mutations cause both cardiomyocyte death, leading to loss of myocardial tissue, and a signal for aberrant repair (Basso et al., 2012), as described further below.

## Molecular mechanisms of ACM pathogenesis

Much basic and translational research activity has been devoted to understanding the mechanisms that underpin ACM pathogenesis (Fig. 1). This research has focused in particular on downstream alterations provoked by causative gene defects. Mutations in the ACM-associated genes that encode desmosomal proteins cause cardiomyocyte connections to be abnormal in shape and composition, and they also cause altered signaling in all the cell types that express the mutated genes (Al-Jassar et al., 2011; Grossmann et al., 2004; Pieperhoff et al., 2008; Bass-Zubek et al., 2009). Importantly, canonical Wnt and Hippo pathways (Box 1) have been recognized to be affected by these desmosomal mutations (Chen et al., 2014).

The Wnt pathway participates in the developmental process during embryogenesis and is implicated in adult tissue homeostasis. In the presence of an active canonical Wnt pathway, β-catenin degradation is inhibited; thus, this protein accumulates in the cytoplasm, translocates into the nucleus and interacts with T-cell factor/lymphoid enhancer factor (TCF/LEF) transcription factors, inducing proliferation and cell fate specification, including cardiomyocyte differentiation (Li et al., 2011). In ACM, the altered metabolism of plakoglobin (PG), a desmosome protein that has a high degree of homology with β-catenin, is thought to be central to disease pathogenesis. In patients with the disease, PG is abnormally found in the nuclear compartment (Garcia-Gras et al., 2006; Sommariva et al., 2016), where it is thought to compete with β-catenin, exerting a detrimental effect on Wnt signaling and ultimately activating adipogenesis, fibrosis and apoptosis (Garcia-Gras et al., 2006).

The Hippo pathway, which responds to cell polarity, diffusible sensing signals and mechanosensing, and which functions to regulate cell proliferation, apoptosis and cell fate, has been also linked to ACM. Yes-associated protein (YAP), the Hippo pathway effector, is phosphorylated in ACM by the Hippo kinase signaling cascade; this phosphorylation inhibits YAP canonical transcriptional activity and consequently limits cellular proliferation. Moreover, YAP interaction with β-catenin further prevents nuclear localization of the latter, inhibiting Wnt and increasing adipogenesis (Chen et al., 2014).

A primarily electrical pathogenic mechanism for ACM has also been proposed. A link between PKP2 and the gap-junction protein connexin 43 (Cx43; encoded by the gene *GJA1*) has been demonstrated (Oxford et al., 2007) based on their coexistence in the same macromolecular complex. This interaction network (‘connexome’) was extended in 2014 to include the voltage-gated sodium channel Nav1.5 (Agullo-Pascual et al., 2014a). The electric current passing through this channel is reduced in cardiac myocytes lacking PKP2 (Sato et al., 2009). The localization of Nav1.5 to cell membranes is Cx43-dependent (Agullo-Pascual et al., 2014b); thus, functionally, Cx43 reduction parallels with Nav1.5 reduction (Jansen et al., 2012; Cerrone and Delmar, 2014). Moreover, the loss of PKP2 stimulates Cx43 complex remodeling, which results in altered intercalated disc (Box 1) structures (Oxford et al., 2007). The loss of PKP2 has been associated with Nav1.5 reduced functionality, both in *PKP2* heterozygous knockout mice (Cerrone et al., 2012) and in Brugada syndrome patients (Box 1), who carry a *PKP2* mutation (Cerrone et al., 2014). Interestingly, the implication of the sodium channel in ACM pathogenesis links Brugada syndrome with ACM; these disorders are already known to share some clinical features, such as RV arrhythmias and sudden death. Of note, a minor structural and functional impairment of the RV, mainly in the RV outflow tract, might also occur in Brugada patients (Pérez Riera et al., 2005; Böhm et al., 2007).

ACM-related mutations are also found in the gene encoding RYR2, a calcium release channel (Tiso et al., 2001), and in the *PLN* gene, which encodes for a membrane protein that regulates the Ca^2+^ pump in cardiac muscle cells (van der Zwaag et al., 2012). This implies that deficient excitation–contraction coupling (Box 1) might contribute to ACM. It has been hypothesized that modifications of intracellular calcium homeostasis contribute to the pathogenesis of ACM by inducing cellular injury, triggering both apoptosis and electrical instability (van der Zwaag et al., 2012; Tiso et al., 2001).

ACM associated with mutation of TMEM43, a transmembrane protein, seems to be mechanistically similar to desmosome-linked ACM, because *TMEM43* mutations lead to PG redistribution, altered Cx43 phosphorylation and function, and impaired conduction velocity (Merner et al., 2008; Siragam et al., 2014). Similar observations have been made by analyzing the effect of *DES* mutations; *DES* encodes desmin, an intermediate filament protein that interacts with DSP. These mutations affect the localization of DSP and PKP2 at the intercalated discs (Otten et al., 2010). Of note, mutations in the *CTNNA3* gene (van Hengel et al., 2013), which encodes for the *area composita* (Box 1) protein αT-catenin, are thought to alter the homodimeric interactions of the protein or its interactions with β-catenin. These data, together with the newest finding of mutations in the *CDH2* gene (Turkowski et al., 2017), coding for N-cadherin, suggest that ACM could be reconsidered to be a disease affecting the *area composita* rather than a purely desmosomal disease.

The association of ACM with mutations of *TGFB3* has been reported (Beffagna et al., 2005). Although a direct causative role has not been proven, this association could consolidate the link between ACM and fibrosis. *TGFB3* encodes for a cytokine that stimulates fibrosis by promoting the expression of extracellular matrix genes and by modulating cell adhesion and the expression of desmosomal genes in different cell types (Kapoun et al., 2004; Yoshida et al., 1992).

Finally, ACM has been linked to mutations in genes coding for the structural proteins titin (stabilizes the sarcomere) and lamin A/C (provides a nuclear-envelope framework and interacts with chromatin), although overlap syndromes cannot be excluded. These mutations lead to an impaired cellular structural stability and a higher protein turnover (Taylor et al., 2011; Forleo et al., 2015), provoking cell death and myocardial remodeling (Box 1). Different *in vitro* models have helped to define the ACM cellular phenotype and to investigate these disease mechanisms.

## Overview of cellular models of ACM

**Fig. 2.**
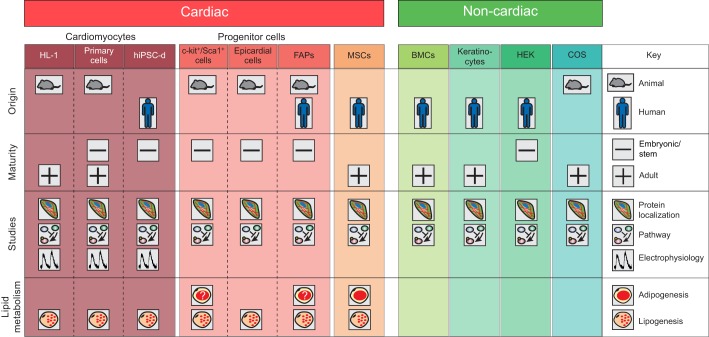
**Cellular models used for *in vitro* studies of ACM.** A schematic illustration of the cardiac and non-cardiac cell models used to study ACM. The figure shows information concerning: the species of origin (animal or human), the stage of cell maturity (adult or embryonic/stem cells), the type of studies performed to date (immunoassays for protein localization, pathway investigation and cellular electrophysiology) and the specific lipid accumulation processes involved (adipo- or lipogenesis). BMCs, buccal mucosa cells; COS, CV-1 in origin carrying the SV40 genetic material, derived from monkey kidney tissue; FAPs, fibro-adipocytes progenitors; HEK, human embryonic kidney 293 cells; hiPSC-d, human induced pluripotent stem cell-derived cardiomyocytes; HL-1, murine immortalized AT-1 atrial cardiomyocytes; MSCs, mesenchymal stromal cells; ?, not clear from the performed investigations.

In this section and in Fig. 2 and Table 1, we give an overview of the *in vitro* models of ACM studied to date. These cell models have contributed to the current understanding of the ACM pathogenic mechanisms explained above. *In vitro* models investigated so far have been derived from the cardiac contractile compartment, progenitor cells, the stromal compartment and non-cardiac cells.

**Table 1.**
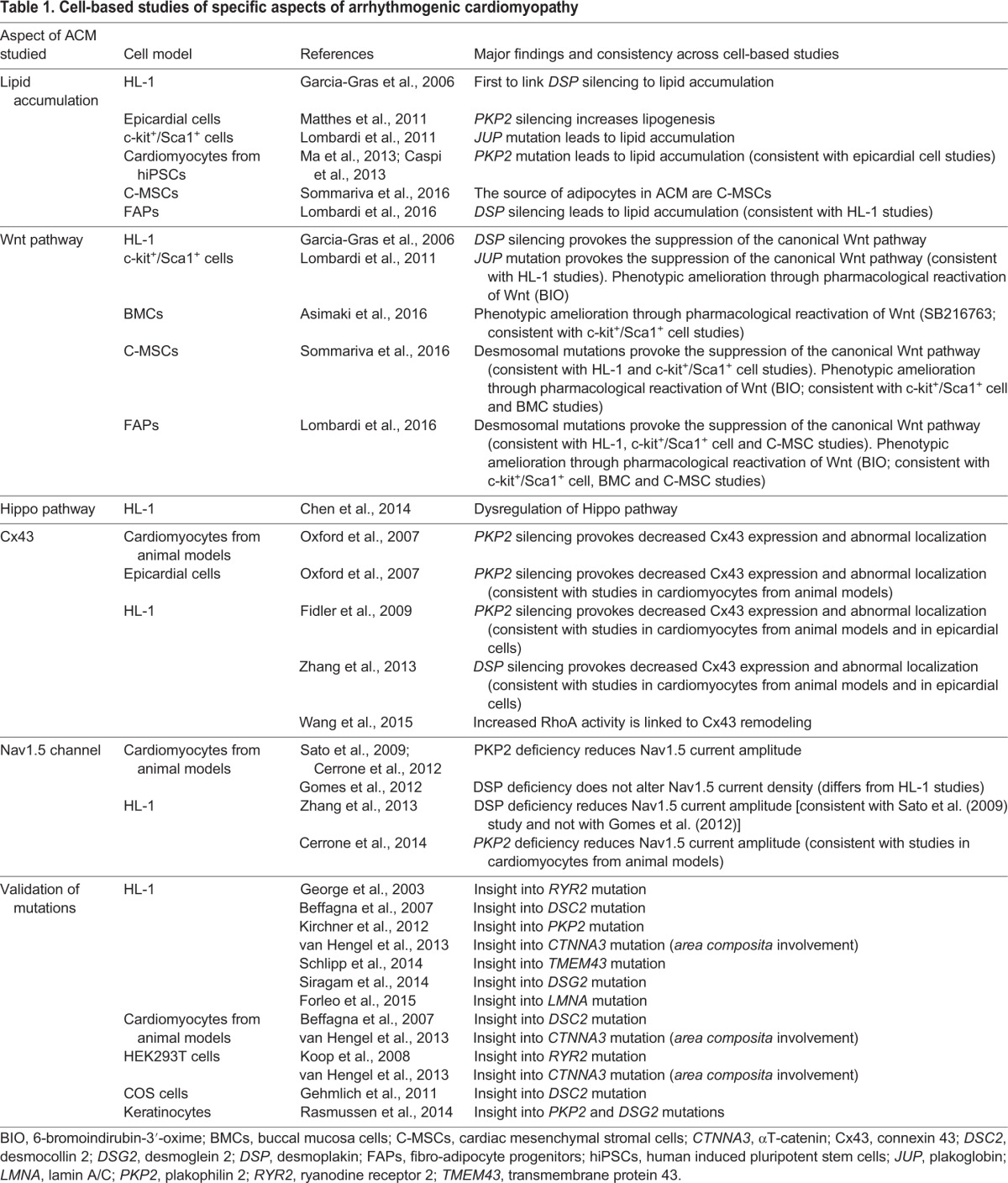
**Cell-based studies of specific aspects of arrhythmogenic cardiomyopathy**

### Cardiomyocytes

Intercalated discs are intercellular specialized areas at the end of cardiomyocytes that enable cardiac muscles to contract in a synchronized manner. They are composed of different kinds of junctions that are essential for myocardial mechanical continuity (via desmosomes), electrical coupling (via gap junctions) and electrical activity (via voltage-gated ion channels) between adjacent cells and, hence, for maintaining correct heart function. In light of the important functions of intercalated discs, cardiomyocytes have been proposed to be the pivotal cellular model in ACM. Adult human cardiomyocytes are, however, difficult to obtain and to maintain in culture; therefore, various surrogates have been used, as we describe below.

#### HL-1 cell line

The HL-1 cell line was obtained with the immortalization of AT-1 atrial cardiomyocytes, isolated from transgenic mice in which expression of the SV40 large T antigen was controlled by the atrial natriuretic factor promoter (Field, 1988). This line was the first cellular model introduced to mimic cardiomyocyte performance in ACM. HL-1 cells contract even after serial passaging, and retain differentiated cardiac morphological, biochemical and electrophysiological properties (Claycomb et al., 1998).

By silencing *DSP* through stable transfection, using siRNA in HL-1 cells, Garcia-Gras et al. demonstrated, for the first time, the translocation of PG into the nucleus, the suppression of the canonical Wnt pathway and an increase in the adipogenic gene expression with a consequent accumulation of lipid droplets (Garcia-Gras et al., 2006). This led to the hypothesis that desmosomal gene knockdown (e.g. *DSP*) might provoke Wnt signaling impairment, possibly mediated by PG. By knocking down *PKP2* in HL-1 cells, Hippo pathway dysregulation was revealed both at the transcript and protein levels, indicating Hippo pathway involvement in ACM pathogenesis (Chen et al., 2014).

The impairment of gap junctions and its effect on electrical synchrony has been demonstrated with the use of *PKP2*-deficient HL-1 myocytes. Consistent with this, Cx43 membrane localization and expression is impaired in these cells (Fidler et al., 2009). A reduced current amplitude of the Nav1.5 sodium channel has also been reported in *PKP2*-deficient HL-1 cells (Cerrone et al., 2014). Wang et al. (2015) recently demonstrated that an increased activity of RhoA can influence Cx43 expression in ACM, providing a potential mechanism to link *PKP2* deficiency to Cx43 remodeling. In addition to PKP2, DSP has also been shown to play an important role in the stability and signaling of the connexome (Zhang et al., 2013). Overall, these findings tell us that mutations in different desmosomal genes result in common impairment of electrical continuity, supporting the theory that this is a direct cause of arrhythmias in ACM.

Studies in HL-1 cells have also helped to link non-desmosomal gene mutations to arrhythmias. A *TMEM43* mutation was found to cause a redistribution of junctional PG and αT-catenin, Cx43 phosphorylation, and altered conduction velocity (Siragam et al., 2014). Another study provided support for the important role of RYR2. Specifically, George et al. (2003) transfected HL-1 cardiomyocytes with a *RYR2* mutated plasmid and reported higher levels of calcium release after stimulation; this affected both the contractile behavior of these cells – possibly leading to the ACM phenotype of cardiac failure – and the cellular repolarization level, thus contributing to the arrhythmic phenotype.

The impact of newly discovered ACM-linked mutations has also been studied in HL-1 cardiomyocytes through the overexpression of the mutated genes. For example, *DSC2* mutations have been studied to define their pathogenicity and evaluate their effect on localization of the mutated protein (Beffagna et al., 2007; De Bortoli et al., 2010; Gehmlich et al., 2011). A *PKP2* missense mutation was shown to generate an unstable PKP2 protein that was incapable of interacting with DSP and was degraded (Kirchner et al., 2012). *DSG2* mutations resulted in a reduced strength of cell–cell contact, demonstrating that DSG2 is crucial for cardiomyocyte cohesion (Schlipp et al., 2014). *LMNA* mutations in HL-1 cells lead to altered nuclear shape and pore organization, which decrease cardiomyocyte adaptation to mechanical stress (Forleo et al., 2015). Interestingly, a role for the inhibitor of apoptosis-stimulating protein of p53 (iASPP) in maintaining the integrity of desmosomes through its interaction with DSP and DES has been modeled in HL-1 cells (Notari et al., 2015). This finding expands the causes of ACM to the regulators of desmosomes as well as desmosomal proteins themselves. Moreover, studies in HL-1 cells were the first to demonstrate the involvement of αT-catenin in ACM (van Hengel et al., 2013), therefore extending the junctional defects of ACM to the *area composita*.

It is important, however, to highlight that the HL-1 line has some notable shortcomings. First, its mouse origins pose a limitation for human disease modeling. Second, HL-1 cells are of atrial derivation, and show an ultrastructure organization that is typical of embryonic atrial cardiac muscle cells, with poorly organized parallel arrays of myofibrils; thus, these cells do not fully recapitulate ventricular cardiomyocytes. This is probably the reason why HL-1 cells are rarely used for electrophysiological studies.

#### Cardiomyocytes from animal models

Both neonatal (to guarantee longer survival in culture) and adult (to better recapitulate adult onset) cardiomyocytes from animal models have been used in ACM research. Two common approaches involve transfecting the cardiomyocytes of wild-type (WT) animals *ex vivo* with ACM-causing mutations or using cardiomyocytes from transgenic animals that carry an ACM-associated mutation.

Consistent with evidence from HL-1 cells, *PKP2* silencing in neonatal rat cardiomyocytes has demonstrated that PKP2 loss causes altered Cx43 levels and distribution (Oxford et al., 2007). In 2009, the same group used *PKP2*-silenced rat cardiomyocytes to show that *PKP2* deficiency affects propagation properties in cardiomyocytes and alters sodium current function (Sato et al., 2009).

WT neonatal rat cardiomyocytes have also been transfected with expression vectors that contain mutations in other genes linked to ACM, including *DSC2* (Beffagna et al., 2007) and *CTNNA3* (van Hengel et al., 2013), and these studies have demonstrated their causative roles in the disease.

Cardiomyocytes from *PG*-knockout mice, studied in parallel with those from a double *PG*/β-catenin knockout, revealed that both of these N-cadherin binding partners are essential for maintaining intercalated disc structure and for mechano-electrical coupling (Swope et al., 2012). Electrophysiological studies have been performed in cardiomyocytes from *PKP2* (Cerrone et al., 2012) and *DSP* (Gomes et al., 2012) heterozygous knockout mice, and reported a deficit or unaltered sodium current density (Box 1), respectively. Although the results with *PKP2* heterozygous knockout mice are in agreement with those obtained in HL-1 cells, the sodium current in *DSP*-knockout murine cardiomyocytes did not show the same impairment as seen in *DSP-*knockdown HL-1 cells.

Transgenic zebrafish models of ACM have also been used to isolate cardiomyocytes for *in vitro* studies. Notably, a transgenic zebrafish with cardiac-specific expression of mutated PG has been used for mechanistic studies, revealing that correct trafficking of intercalated disc proteins is crucial for cardiomyocyte integrity (Asimaki et al., 2014; Macrae, 2010).

Even if animal-derived cardiomyocytes represent a valuable and accessible source of functional cells, they suffer the limitation of their non-human origin. Consequently, insights obtained with these tools still need to be confirmed in human-derived models.

#### Cardiomyocytes from induced pluripotent stem cells

Human induced pluripotent stem cells (hiPSCs) represent a tool to obtain human-derived cardiomyocytes (Brandão et al., 2017) and so overcome the interspecies issues noted above. The first hiPSC-derived ACM cardiomyocytes were generated in 2013 using skin fibroblasts from an ACM patient. These cells showed reduced *PKP2* and *PG* expression and an increased lipid-droplet accumulation when cultured in adipogenic differentiation medium (Ma et al., 2013).

One of the unsolved shortcomings of hiPSC-derived cardiomyocytes is their fetal-like phenotype (Ma et al., 2013), which does not fully recapitulate the adult cardiomyocyte. Kim et al. (2013) partially overcame this issue by inducing an adult-like metabolism in ACM-hiPSC cardiomyocytes by stimulating fatty-acid oxidation. Subsequently, desmosomal ultrastructural changes were studied in cardiomyocytes differentiated from ACM-hiPSCs, identifying a correlation between the extent of desmosomal structural abnormalities and predisposition to lipid accumulation (Caspi et al., 2013).

Despite well-known technical limitations, including a high variability among clones obtained from the same donor, the advantages of hiPSCs as ACM cell models include their human origin, their potential unlimited availability and their suitability for high-throughput screening. Moreover, they carry patient genomes, representing a unique tool for personalized-medicine approaches. It is also worth noting that, to date, no studies have attempted to use genome editing to correct ACM-associated mutations in ACM-hiPSCs, which would provide indisputable proof of phenotype–genotype coupling.

### Progenitor cells

Progenitor cells have been used to model ACM because of their stem-cell-like multipotency, and their higher adipogenic potential compared to terminally differentiated cells.

#### c-kit^+^/Sca1^+^ murine cells

c-kit^+^/Sca1^+^ murine cells have been used as an ACM cell model. c-kit^+^ cells are the first resident stem-cell population identified in the heart (Beltrami et al., 2003). These cells are self-renewing and multipotent *in vitro*, and can repair damaged myocardium (Dey et al., 2013). They can also accumulate fat upon *in vitro* adipogenic stimulation (Gambini et al., 2010). Sca1 identifies a heterogeneous population of adult cells, including endothelial, stromal and vascular cell progenitors. Cells expressing Sca1 show typical features of stem cells, are characterized by cardiogenic potentials (Oh et al., 2003) and can accumulate fat *in vitro* (Matsuura et al., 2004). *JUP*-overexpressing c-kit^+^/Sca1^+^ cells have been obtained from transgenic mouse hearts and used to show that PG translocation into the nucleus and the consequent repression of Wnt/β-catenin lead to adipogenic differentiation (Lombardi et al., 2011). This study, in accordance with previous findings, helped to demonstrate that PG is an essential mediator of the myogenesis-to-adipogenesis switch, and that adipocytes in ACM patients' hearts originate, at least in part, from c-kit^+^/Sca1^+^ cells (Lombardi et al., 2011). However, this last finding has been questioned by a recent study that provided evidence of few c-kit^+^ cells differentiating in adipocytes in ACM hearts (Sommariva et al., 2016). Lately, even the cardiomyogenic potential of c-kit^+^ cells is a matter of debate (van Berlo et al., 2014).

A key shortcoming of these progenitors is that they are difficult to obtain by cell sorting, and they represent a very small subpopulation of cardiac cells. It is also questionable whether they are all resident cells. Finally, the c-kit^+^/Sca1^+^ cells extensively studied were of mouse origin.

#### Epicardial cells

Epicardial cells compose the epithelial monolayer surrounding the heart. They exert both a protective role and a functional role in the myocardial response to injury. Epithelial cells share a common origin with second heart field (Box 1) progenitors (Zhou et al., 2008), and have been used to model ACM. The silencing of *PKP2* in epicardial progenitors from WT neonatal rat hearts causes changes in Cx43 amount and distribution (Oxford et al., 2007). In line with other data, PKP2 and Cx43 coexist in the same macromolecular complex in epicardial cells (Oxford et al., 2007). Subsequently, the role of PKP2 in the migration, proliferation and transdifferentiation of cultured primary epicardial cells was studied, and demonstrated that increased lipogenesis and myofibroblast differentiation could be related to *PKP2* loss. Therefore, it was theorized that epicardial and epicardial-derived cells can act as adipocyte progenitors and contribute to fibrosis (Matthes et al., 2011). Further studies are needed to provide robust proof of this hypothesis.

#### Fibro-adipocyte progenitors

Fibro-adipocyte progenitors (FAPs) represent an alternative cellular source for investigating fat and fibrosis accumulation in ACM (Lombardi et al., 2016). FAPs are resident skeletal-muscle progenitor cells, characterized by the platelet-derived growth factor receptor α (PDGFRα) marker. FAPs seem to be bi-potential: different subpopulations express fibroblast markers or adipogenic transcription factors. Using this model, Lombardi et al. (2016) confirmed that DSP deficiency suppresses Wnt signaling, and that this effect is ameliorated through Wnt pharmacological reactivation. Moreover, adipocyte proliferation was excluded in ACM, in favor of the hypothesis of FAP differentiation through the activation of adipogenic transcription factors.

### Non-cardiac cells

#### Buccal mucosa cells

Owing to the limited availability of human myocardial samples, Asimaki et al. (2016) have proposed buccal mucosa cells (BMCs) as an *in vitro* model of ACM. BMCs, which are obtained easily from the inside of the mouth, are epithelial cells; thus, they express gap junctions and desmosomes, like cardiac cells. The authors studied the distribution of proteins usually present in intercalated discs (e.g. PG and Cx43) and found the same altered distribution in ACM-patient-derived BMCs as in the patient cardiac tissue. They also showed that Wnt pharmacological reactivation can apparently restore normal PG and Cx43 localization in ACM cells (Asimaki et al., 2016). Thus, despite their non-cardiac derivation, these cells might be an additional useful tool that can be easily obtained from large numbers of ACM patients at minimal cost to investigate disease mechanisms and use in drug screening.

#### Primary keratinocytes

Primary keratinocytes are another easily obtainable adult human-derived cell type that express high levels of all isoforms of desmosomal proteins (Gerull, 2014). Rasmussen et al. (2014) showed that changes in myocardial expression of PKP2 and DSG2 are mirrored by similar changes in keratinocytes (Rasmussen et al., 2014). These findings suggest that, despite being of non-cardiac origin, these keratinocytes might represent a new accessible source of cells to model patient-specific ACM mutations.

#### HEK293T cells

The HEK293T cell line, originally derived from human embryonic kidney, has been used to conduct functional tests on a newly identified ACM-associated genetic mutation in *CTNNA3*. Transfection of the mutant gene into this cell line revealed that the interaction between mutant αT-catenin and β-catenin was weaker than with WT αT-catenin (van Hengel et al., 2013). This cell model, which naturally lacks endogenous RYR2 channels, has also been used to study the effect of two *RYR2* mutations on the store-overload-induced calcium-release activity. This study found that the combination of the two *RYR2* mutations, which affect important residues for RYR2 tetramer formation and function, caused significant changes in calcium release activity (Koop et al., 2008). By identifying the additive effect of the two mutations, this study revealed the reason why carriers of compound heterozygous mutations in the *RYR2* gene can be affected by ACM.

#### COS cells

COS cells, immortalized cell lines derived from monkey kidney tissue, have also been used to investigate functional impairments caused by ACM-associated mutations. Rajkumar et al. (2012) used COS-7 cells to study the localization of WT and mutated TMEM43; no change was observed in desmosomal stability or in the localization of TMEM43 and two of its binding partners, lamin B and emerin, in the presence of mutated TMEM43 (Rajkumar et al., 2012). Finally, COS-1 cells have been used to evaluate the role of *DSC2* mutations in causing ACM. An impaired maturation of mutated DSC2 was observed, along with a reduced binding to PG (Gehmlich et al., 2011). This finding is in accordance with the reduced localization of PG at desmosomes in intercalated discs of ACM patients' heart tissue, reported earlier by the same authors (Asimaki et al., 2009). This model, however, suffers different limitations: not only are these cells not cardiac, but they are also of animal origin.

### Adult cardiac stromal cells

In 2015, we proposed non-contractile cardiac mesenchymal stromal cells (C-MSCs) as a novel cell model for ACM (Sommariva et al., 2016). These cells are abundant in the heart and are involved in maintaining cardiac cell structure and functional homeostasis in physiological and pathological conditions (Brown et al., 2005). In 2010, C-MSCs were isolated from human adult auricles (Box 1) and characterized for the first time (Rossini et al., 2010). C-MSCs are primary cells obtained directly from human cardiac tissue after enzymatic digestion with collagenase and selection for plastic adherence, and they express typical mesenchymal markers (CD29, CD105, CD44, CD90) (Rossini et al., 2010). Like their bone-marrow counterpart (BM-MSCs), C-MSCs can differentiate in endothelium, osteocytes and adipocytes (Rossini et al., 2010), and are more likely than BM-MSCs to express cardiovascular lineage markers upon cardiogenic stimulus. They can also be easily amplified and maintained *in vitro* for many passages. Notably, C-MSCs carry patient-specific mutations and their genetic background (Sommariva et al., 2016). We demonstrated, for the first time, that, in the explanted hearts of ACM patients, C-MSCs are involved in active adipogenic differentiation (Sommariva et al., 2016). C-MSCs isolated from patient ventricular biopsies express desmosomal genes and, when cultured in adipogenic medium, are more prone to differentiate into adipocytes than are control C-MSCs (Sommariva et al., 2016). We took advantage of this cell model to confirm some of the above-mentioned molecular mechanisms of ACM, such as PG nuclear localization. Moreover, C-MSCs were used to demonstrate that ACM-specific features are dependent on PKP2 deficiency and on Wnt pathway mis-regulation (Sommariva et al., 2016). In conclusion, C-MSCs represent another promising new cell model for *in vitro* studies of ACM mechanisms.

## Current cell models of ACM: pros and cons

As highlighted in the previous section, different molecular mechanisms of ACM have been investigated *in vitro* thanks to the availability of several cell models. Below, we aim to point out the ‘lights and shadows’ of each cell type, to help guide researchers who want to focus on a specific aspect of ACM. Each *in vitro* model described has intrinsic advantages and disadvantages, depending on its origin (animal versus human; cardiac versus non-cardiac), its maturity (embryonal or undifferentiated versus adult or fully differentiated) and on the cell type (parenchymal versus stromal). On the basis of these features, and taking together the findings summarized in Table 1 and Fig. 2, it is possible to choose which should be the most suitable cell type in which to investigate specific aspects of ACM.

ACM mouse models do not show the extensive cardiac adipose deposits typical of ACM patients (Cerrone et al., 2012; Krusche et al., 2011), and so cell-based models are preferable to investigate causative pathways linked to lipid metabolism. Adult cells of human cardiac origin, carrying patient-specific mutations and genetic backgrounds, represent the best tool for these studies. However, adult cardiomyocytes are of limited accessibility and must be obtained by invasive sampling. Moreover, they do not replicate, are difficult to maintain in culture and impose the constraint of restricted transdifferentiation potential. Indeed, manifest lipid accumulation has been reported only in immature cardiomyocyte models, such as those obtained from hiPSCs, which still possess a residual potency (Kim et al., 2013). Also, progenitor cells do differentiate easily *in vitro*, but this could possibly be related more to their multipotency than to disease-specific differentiation (Lombardi et al., 2011; Matthes et al., 2011). To overcome these limitations, C-MSCs represent the ideal model for studying lipid metabolism, because they undergo adipogenic differentiation in patient hearts and maintain the same ability *in vitro* (Sommariva et al., 2016).

Because electrical activity is restricted to cardiomyocytes, these provide the only eligible models to investigate gap-junction and ion-channel localization, and for electrophysiological studies: the cardiomyocytes can be either primary or immortalized cells obtained from animal models or from patient hiPSCs. To date, limited electrophysiological data are available on the latter (Kim et al., 2013), although hiPSC-derived cardiomyocytes are potentially the best model to recapitulate the human pathological scenario, with the limitation of a fetal-like phenotype (meaning that adult-onset disease cannot be mimicked). Moreover, further studies on hiPSC-derived cardiomyocytes could help to shed further light on different currents, including sodium current, which have been studied in murine-derived cell models, with conflicting results (Gomes et al., 2012; Zhang et al., 2013).

Despite their non-cardiac derivation, BMCs represent a possible tool for large population studies because the sampling technique is not invasive. Other non-cardiac (and easily accessible) cells in which desmosomes are expressed could eventually be considered in the future.

Finally, functional validation of mutations often relies on overexpression of plasmids carrying mutated genes. Undoubtedly, it is preferable to use patient-derived cells that already carry the desired mutations, such as primary cells or hiPSC-derived cardiomyocytes. However, the presence of the patient's whole genetic background, including main mutations and modifier variants, might make it difficult to assign single-variant pathogenicity. To overcome this issue and to enable a direct comparison between mutated and WT cells, the more controlled tool of cardiomyocytes from transgenic animals have been used (Beffagna et al., 2007; van Hengel et al., 2013). The newest biomolecular techniques, such as CRISPR/Cas9 will now allow direct correction of mutations in human cells, thus allowing direct comparison between mutated and corrected cells.

## Unanswered questions and future perspectives

Despite the advances made to date, many questions about ACM pathogenesis remain unanswered, and cell models could help to shed light on these outstanding questions.

First, the genetic causes of ACM are not yet fully known. Notably, about 50% of the probands undergoing genetic screening fail to have a causative mutation in ACM-associated genes (Marcus et al., 2013). ACM is characterized by high phenotypic variability and low penetrance, and, in some patients, by compound heterozygosity (Lazzarini et al., 2015). Moreover, a screening of ACM-associated genetic variants in a population of healthy individuals resulted in 18% positivity rate, questioning the causative role of these variants (Andreasen et al., 2013). This evidence indicates that considerable genetic heterogeneity is involved in ACM and that it might not be a monogenic Mendelian disorder. Moreover, non-genetic cofactors might also contribute to ACM pathogenesis, either providing the trigger for disease development or worsening disease severity. A deeper understanding of ACM-associated genes and cofactors might help to unravel the contribution of new pathways, or to refine our understanding of other known molecular mechanisms. Direct comparisons of desmosomal versus non-desmosomal gene-associated mutations are needed in the same cell type to better understand downstream disease mechanisms. Given the complexity of ACM genetics, the identification of digenic inheritance (Xu et al., 2010) and the contribution of modifier alleles (Sen-Chowdhry et al., 2010), it would be interesting to investigate cell models modified with more than one ACM-associated mutation. Alternatively, primary cells, carrying the entire genetic background of an ACM patient, could be compared with controls in which the disease variant is selectively corrected. These cells will help to unravel the specific contribution of what is considered the main causative mutation, with respect to the pathogenicity of the other variants. Interestingly, potential mosaicisms or somatic mutations (Lubitz and Ellinor, 2015) have never been investigated in ACM. Human cardiac primary cells are suitable for this.

Certain ACM features are difficult to recapitulate *in vitro*. Specific studies are needed to understand the causes of adult-onset ACM and male prevalence. In long QT (Box 1) (Salama and Bett, 2014) and Brugada (Benito and Berruezo, 2014) syndromes, the steroid profile is thought to provoke post-puberty cardiac electrical-property changes. ACM gender differences deserve specific mechanistic studies using male versus female models or investigations about the effect of hormones on the ACM cellular phenotypes. In addition, the reason for the association between intense physical activity and ACM risk (Saberniak et al., 2014) is not known. It has been hypothesized that strong mechanical stretch during exercise acts on the RV, leading to myocardial damage and promoting cardiac remodeling. Furthermore, intense sport provokes sympathetic stimulation, which is a known trigger of arrhythmias (Shen and Zipes, 2014). Indeed, impairment of cardiac sympathetic innervation and a significant reduction of postsynaptic β-adrenergic density have been described in ACM patients (Wichter et al., 2000). In this context, it would be interesting to address the cellular effect of mechanical or chemical stimulation. Moreover, the preponderance of RV myocardial remodeling in ACM has not yet been fully explained. A tentative response to this question has been provided by evidence that the cellular developmental origin of pre-adipocytes in ACM hearts is the second heart field (Lombardi et al., 2009; Zhou et al., 2008), which gives rise to the RV. Alternatively, the physiological difference between RV and left ventricle (LV) thickness and wall tension might provoke a different mechanotransduction of ACM signaling (Thiene and Marcus, 2013). Modeling ACM with RV versus LV cells will help to reveal area-of-origin-specific mechanisms.

One of the most appealing applications of reliable cell models is for high-throughput drug screening and/or candidate testing. To date, a high-throughput screen performed in a zebrafish ACM model has identified the compound SB216763 as a disease phenotype suppressor (Asimaki et al., 2014). No ACM cell model has hitherto been used for drug screening. Marian and coworkers tested one candidate, GSK3β inhibitor 6-bromoindirubin-3′-oxime (BIO), in c-Kit^+^/Sca1^+^ cells isolated from the heart of mice overexpressing truncated PG and obtained Wnt pathway restoration and phenotypic rescue (Lombardi et al., 2011). BIO and SB216763 have been largely used thereafter in ACM cell models to verify Wnt pathway involvement (Asimaki et al., 2016; Sommariva et al., 2016; Lombardi et al., 2016; Kim et al., 2013; Caspi et al., 2013; Hariharan et al., 2014).

The heart is a complex integrated network, composed of qualitatively and quantitatively different cell types, including, among others, myocytes, stromal cells, fibroblasts, adipocytes, smooth muscle cells, endothelial cells and pericytes, which are finely tuned through direct and paracrine interactions. New multicellular models will likely be needed to understand the interplay between the myocyte and non-myocyte compartments and how they singularly or synergistically contribute to the different aspects of ACM pathogenesis. Moreover, tissue-engineered scaffolds that mimic the myocardial three-dimensional structure will help to create complex cellular models that allow in-depth mechanistic assessments as well as tissue-level validation of the effect of novel therapeutic compounds.
